# Consolidating the set of known human protein-protein interactions in preparation for large-scale mapping of the human interactome

**DOI:** 10.1186/gb-2005-6-5-r40

**Published:** 2005-04-15

**Authors:** Arun K Ramani, Razvan C Bunescu, Raymond J Mooney, Edward M Marcotte

**Affiliations:** 1 Center for Systems and Synthetic Biology and Institute for Cellular and Molecular Biology, University of Texas, Austin, TX 78712, USA; 2 Department of Computer Sciences, University of Texas, Austin, TX 78712, USA; 3 Department of Chemistry and Biochemistry, University of Texas, Austin, TX 78712, USA

## Abstract

**Background:**

Extensive protein interaction maps are being constructed for yeast, worm, and fly to ask how the proteins organize into pathways and systems, but no such genome-wide interaction map yet exists for the set of human proteins. To prepare for studies in humans, we wished to establish tests for the accuracy of future interaction assays and to consolidate the known interactions among human proteins.

**Results:**

We established two tests of the accuracy of human protein interaction datasets and measured the relative accuracy of the available data. We then developed and applied natural language processing and literature-mining algorithms to recover from Medline abstracts 6,580 interactions among 3,737 human proteins. A three-part algorithm was used: first, human protein names were identified in Medline abstracts using a discriminator based on conditional random fields, then interactions were identified by the co-occurrence of protein names across the set of Medline abstracts, filtering the interactions with a Bayesian classifier to enrich for legitimate physical interactions. These mined interactions were combined with existing interaction data to obtain a network of 31,609 interactions among 7,748 human proteins, accurate to the same degree as the existing datasets.

**Conclusion:**

These interactions and the accuracy benchmarks will aid interpretation of current functional genomics data and provide a basis for determining the quality of future large-scale human protein interaction assays. Projecting from the approximately 15 interactions per protein in the best-sampled interaction set to the estimated 25,000 human genes implies more than 375,000 interactions in the complete human protein interaction network. This set therefore represents no more than 10% of the complete network.

## Abstract 2

In order to consolidate the known human proteins interactions two tests were developed to measure the relative accuracy of the available interaction data. In addition, 6,580 interactions among 3,737 human proteins were recovered from Medline abstracts and combined with existing interaction data to obtain a network of 31,609 interactions among 7,748 human proteins, accurate to the same degree as the existing data sets.

## Background

The past few years have seen a tremendous development of functional genomics technologies. In particular, the yeast proteome has been the subject of considerable effort, including genome-wide protein interaction assays using yeast two-hybrid technology [1,2], affinity chromatography/mass spectrometry [3,4], synthetic lethal assays [5,6], and genome context methods [7-10]. Success in these areas, even given the limited accuracy of these technologies [11-15], has led to the application of the yeast two-hybrid method for the fly [16] and the worm proteomes [17], providing initial steps toward maps of the fly and worm interactomes.

Only minimal progress has been made with respect to the human proteome. The existing protein interaction data are largely composed of small-scale experiments collected in the BIND [18] and DIP [19] databases, as well as a set of approximately 12,000 interactions recovered by manual curation from Medline articles [20] and interactions transferred from other organisms on the basis of orthology [21]. The Reactome database [22] has around 11,000 interactions [23] that have been manually entered from articles focusing on core cellular pathways. Large-scale interaction assays among human proteins have yet to be performed, although a medium-scale map was created for the purified TNFα/NFκB protein complex [24] and the proteins involved in the human Smad signaling pathway [25]. This situation is in stark contrast to the abundant data available for yeast and calls for the application of high-throughput interaction assays for mapping the human protein interaction network.

One lesson from the yeast interactome research is clear: it is critical that such upcoming interaction assays be accompanied by measured error rates, without which the utility and interpretability of the data is jeopardized. To establish a basis for future interaction mapping we sought to consolidate existing human protein interaction data and to establish quantitative tests of data accuracy. We also sought to use data-mining approaches to extract additional known interactions from Medline abstracts to add to the existing interactions.

Most of the current biological knowledge can be retrieved from the Medline database, which now has records from more than 4,800 journals accounting for around 15 million articles. These citations contain thousands of experimentally recorded protein interactions. However, retrieving these data manually is made difficult by the large number of articles, all lacking formal structure. Automated extraction of information would be preferable, and therefore, mining data from Medline abstracts is a growing field [26-29].

In this paper, we present two quantitative tests (benchmarks) of the accuracy of large-scale human protein interaction assays, test the existing sets of interaction data for their relative accuracy, then apply these benchmarks in order to recover protein interactions from the approximately 750,000 Medline abstracts that concern human biology, resulting in a set of 6,580 interactions between 3,737 proteins of accuracy comparable to manual extraction. Combination of the interaction data creates a consolidated set of 31,609 interactions between 7,748 human proteins. On the basis of this initial set of interactions, we estimate the scale of the human interactome.

## Results

### Assembling existing public protein interaction data

We first gathered the existing human protein interaction datasets (summarized in Table 1), representing the current status of the human interactome. This required unification of the interactions under a shared naming and annotation convention. For this purpose, we mapped each interacting protein to LocusLink (now EntrezGene) identification numbers and retained only unique interactions (that is, for two proteins A and B, we retain only A-B or B-A, not both. We have chosen to omit self-interactions, A-A or B-B, for technical reasons, as their quality cannot be assessed on the functional benchmark we develop). In most cases, a small loss of proteins occurs in the conversion between the different gene identifiers (for example, converting from the NCBI 'gi' codes in BIND to LocusLink identifiers). In the case of the Human Protein Reference Database (HPRD), this processing resulted in a significant reduction in the number of interactions from 12,013 total interactions to 6,054 unique, non-self interactions, largely due to the fact that HPRD often records both A-B and B-A interactions, as well as a large number of self interactions, and indexes genes by their common names rather than conventional database entries, often resulting in multiple entries for different synonyms.

**Figure 1:**
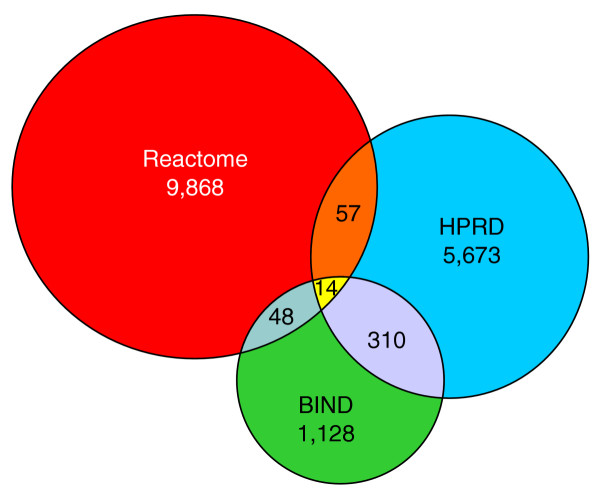
Overlap between existing human protein interaction sets. A Venn diagram shows the overlap is small among the existing, publicly available human protein interaction datasets (specifically, Reactome, BIND, and HPRD protein interaction data). The small overlap (< 0.1% in common in all three datasets) implies that the number of protein interactions described in the literature is actually quite large and that the individual datasets carry specific biases.

Although the interactions from these datasets are in principle derived from the same source (Medline), the sets are quite disjoint (Figure 1), implying either that the sets are biased for different classes of interactions, or that the actual number of interactions in Medline is quite large. We suspect both reasons. It is clear that each dataset has a different explicit focus (Reactome towards core cellular machinery, HPRD towards disease-linked genes, and BIND more randomly distributed). Due to these biases, it is likely that many interactions from Medline are still excluded from these datasets. The maximal overlap between interaction datasets is seen for BIND: 25% of these interactions are also in HPRD or Reactome; only 1% of Reactome interactions are in HPRD or BIND. An additional 9,283 (or around 60,000 at lower confidence) interactions are available from orthologous transfer of interactions from large-scale screens in other organisms (orthology-core and orthology-all) [21].

### Benchmarking of protein interaction data

To measure the relative accuracy of each protein interaction dataset, we established two benchmarks of interaction accuracy, one based on shared protein function and the other based on previously known interactions. First, we constructed a benchmark in which we tested the extent to which interaction partners in a dataset shared annotation, a measure previously shown to correlate with the accuracy of functional genomics datasets [13,14,21]. We used the functional annotations listed in the Kyoto Encyclopedia of Genes and Genomes (KEGG) [30] and Gene Ontology (GO) [31] annotation databases. These databases provide specific pathway and biological process annotations for approximately 7,500 human genes, assigning human genes into 155 KEGG pathways (at the lowest level of KEGG) and 1,356 GO pathways (at level 8 of the GO biological process annotation). KEGG and GO annotations were combined into a single composite functional annotation set, which was then split into independent testing and training sets by randomly assigning annotated genes into the two categories (3,792 and 3,809 annotated genes respectively). For the second benchmark based on known physical interactions, we assembled the human protein interactions from Reactome and BIND, a set of 11,425 interactions between 1,710 proteins. Each benchmark therefore consists of a set of binary relations between proteins, either based on proteins sharing annotation or physically interacting. Generally speaking, we expect more accurate protein interaction datasets to be more enriched in these protein pairs. More specifically, we expect true physical interactions to score highly on both tests, while non-physical or indirect associations, such as genetic associations, should score highly on the functional, but not the physical interaction, test.

For both benchmarks, the scoring scheme for measuring interaction set accuracy is in the form of a log odds ratio of gene pairs either sharing annotations or physically interacting. To evaluate a dataset, we calculate a log likelihood ratio (LLR) as:



where P(D|I) and P(D|~I) are the probability of observing the data (D) conditioned on the genes sharing benchmark associations (I) and not sharing benchmark associations (~I). By Bayes theorem, this equation can be rewritten as:



where P(I|D) and P(~I|D) are the frequencies of interactions observed in the given dataset (D) between annotated genes sharing benchmark associations (I) and not sharing associations (~I), respectively, while P(I) and P(~I) represent the prior expectations (the total frequencies of all benchmark genes sharing the same associations and not sharing associations, respectively). This latter version of the equation is simpler to compute. A score of zero indicates interaction partners in the dataset being tested are no more likely than random to belong to the same pathway or to interact; higher scores indicate a more accurate dataset.

Among the literature-derived interactions (Reactome, BIND, HPRD), a total of 17,098 unique interactions occur in the public datasets. Testing the existing protein interaction data on the function benchmark reveals that Reactome has the highest accuracy (LLR = 3.8), followed by BIND (LLR = 2.9), HPRD (LLR = 2.1), core orthology-inferred interactions (LLR = 2.1) and the non-core orthology-inferred interaction (LLR = 1.1). The two most accurate datasets, Reactome and BIND, form the basis of the protein interaction-based benchmark. Testing the remaining datasets on this benchmark (that is, for their consistency with these accurate protein interaction datasets) reveals a similar ranking in the remaining data. Core orthology-inferred interactions are the most accurate (LLR = 5.0), followed by HPRD (LLR = 3.7) and non-core orthology inferred interactions (LLR = 3.7).

### Recognizing protein names with a conditional random field (CRF) algorithm

To expand the list of human interactions, we turned to literature mining. We adopted the strategy of separately identifying the protein names in the abstracts and then matching up the interacting protein partners. This process was made difficult by the fact that unlike other organisms, such as yeast or *Escherichia coli*, the human genes have no standardized naming convention, and thus present one of the hardest sets of gene/protein names to extract. For example, human proteins may be named with typical English words, such as 'light', 'map', 'complement', and 'Sonic Hedgehog'. Names may be alphanumeric, may include Greek or Roman letters, may be case sensitive, and may be composed of multiple words. Names are frequently sub-strings of each other, such as 'epidermal growth factor' and 'epidermal growth factor receptor', which refer to two distinct proteins. It is therefore necessary that an information-extraction algorithm be specifically trained to extract gene and protein names accurately.

**Figure 2:**
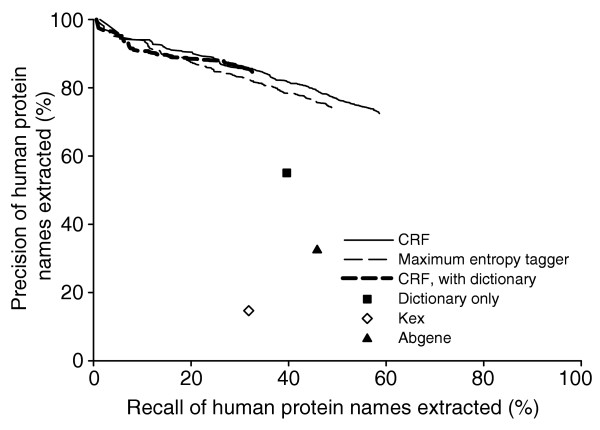
Comparison of precision and accuracy of the algorithms. The conditional random fields (CRF) algorithm considerably outperforms other approaches for identifying human protein names in Medline abstracts, such as the simple matching of words to a dictionary of protein names, as well as the other available protein name-tagging algorithms in [32], Kex [34] and Abgene [35]. The tests are performed on 200 manually annotated Medline abstracts [33]. The precision (the number of correct protein names among all identified names) in identifying proteins is plotted against the recall (the number of correct protein names among all possible correct protein names). Higher scores on both precision and recall are preferable; however, for this purpose, we seek to maximize precision and can tolerate lower recall.

We developed an algorithm capable of distinguishing human protein names from similar words on the basis of their context in the sentence. Building on our previous work in this area [32], we developed a classification algorithm that accurately recognized human protein names in Medline abstracts. The performance of the protein name 'tagger' on a set of human-labeled test abstracts is plotted in Figure 2. The accuracy of the algorithm was measured as its precision (the fraction of correct protein names identified among all identified names) and its recall (the fraction of correctly identified protein names among all possible correct protein names) on a set of 200 publicly available hand-tagged abstracts [33] as well as on 750 Medline abstracts with hand-labeled human protein names (comparable results; data not shown). The algorithm, termed the CRF algorithm due to its use of conditional random fields, significantly out-performs the picking of exact protein names from a dictionary ('dictionary only') by taking into account the words' parts of speech and the context in which they appear. The CRF algorithm also outperforms the other name recognition algorithms available in the public domain [32,34,35]. To prepare for extracting protein interactions, the names of human proteins were identified using the CRF algorithm in the complete set of 753,459 Medline abstracts citing the word 'human'.

### Extracting functional interactions via co-citation analysis

**Figure 3:**
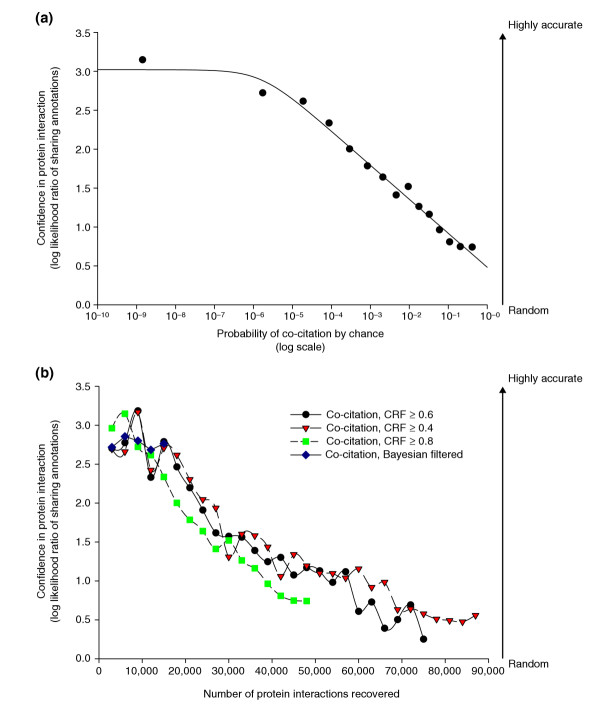
The performance of the co-citation algorithm at identifying protein interactions. **(a) **The probabilistic score effectively ranks co-cited proteins by their tendency to participate in the same pathway, as measured on the functional annotation training benchmark. As the probability of random co-citation decreases, the functional relatedness of the co-cited proteins increases. This tendency is robust to changes in the CRF confidence threshold chosen (data not shown). Each point represents 3,000 protein pairs. **(b) **An examination of the number of protein pairs identified at different CRF thresholds (0.8, 0.6, and 0.4) shows that the recall of the method is increased with lowered thresholds. Re-ranking the 15,000 top-scoring protein pairs (CRF threshold = 0.8) by the tendency of the abstracts to discuss physical protein interactions shows their consistent performance in the annotation benchmark.

In order to establish which interactions occurred between the proteins identified in the Medline abstracts, we used a two-step strategy: measure co-citation of protein names, then enrich these pairs for physical interactions using a Bayesian filter. First, we counted the number of abstracts citing a pair of proteins, and then calculated the probability of co-citation under a random model. Figure 3a shows the performance of the co-citation algorithm, plotting the probability of being co-cited by random chance against the accuracy, calculated as a log likelihood score based on the functional annotation training benchmark. Empirically, we find the co-citation probability has a hyperbolic relationship with the accuracy on this benchmark, with protein pairs co-cited with low random probability scoring high on the benchmark.

The co-citation algorithm is remarkably robust to variations in the minimal accuracy with which the protein names are identified by the CRF algorithm (Figure 3b). This robustness is presumably due to the fact that co-citation requires proteins to be named repeatedly across many abstracts, thereby tolerating occasional errors in the name extraction process. With a threshold on the estimated extraction probability of 80% (as computed by the CRF model) in the protein name identification, around 15,000 interactions are extracted with the co-citation approach that score comparably or better on the independent functional annotation test benchmark than the manually extracted interactions from HPRD, which serves to establish a minimal threshold for our mined interactions.

**Figure 4:**
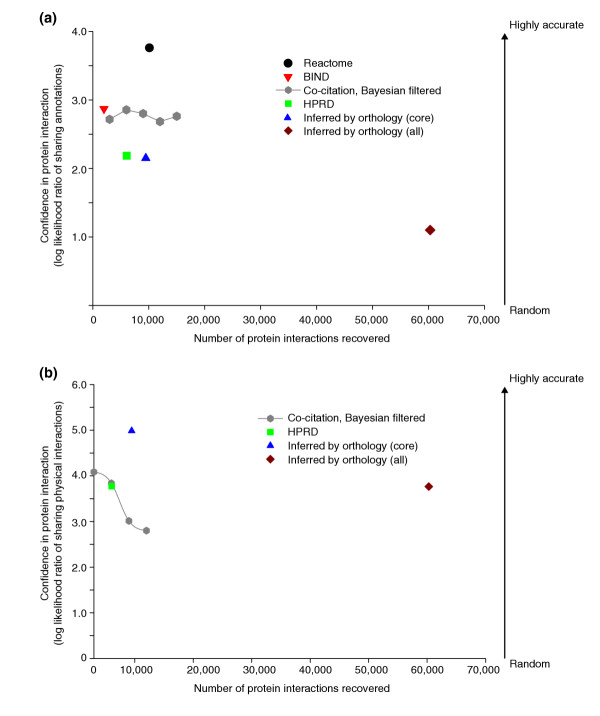
A comparison of the available human protein interaction data on the two benchmarks. **(a) **An examination of the initial performance of the datasets on the functional annotation test benchmark reveals the relative quality of each dataset. The interactions extracted using co-citation analysis filtered by the Bayesian estimator show a robust behavior in terms of their scores. **(b) **Comparison of the performance of the interactions retrieved from the co-citation analysis after incorporating the Bayesian filter and the interactions from HPRD and orthology transfer, as assessed on the physical interaction benchmark. The Bayesian filter effectively ranks the co-citation-derived interactions in terms of their correspondence to physical protein interactions.

However, it is clear that proteins are co-cited for many reasons other than physical interactions. We therefore tried to enrich specifically for physical interactions by applying a secondary filter: We applied a Bayesian classifier to measure the likelihood of the abstracts citing the protein pairs to discuss physical protein-protein interactions. The classifier [36] scores each of the co-citing abstracts according to the usage frequency of words relevant to physical protein interactions. Interactions extracted by co-citation and filtered using the Bayesian estimator compare favorably with the other interaction datasets on the functional annotation test benchmark (Figure 4a). Testing the accuracy of these extracted protein pairs on the physical interaction benchmark (Figure 4b) reveals that the co-cited proteins scored high by this classifier are indeed strongly enriched for physical interactions.

**Figure 5:**
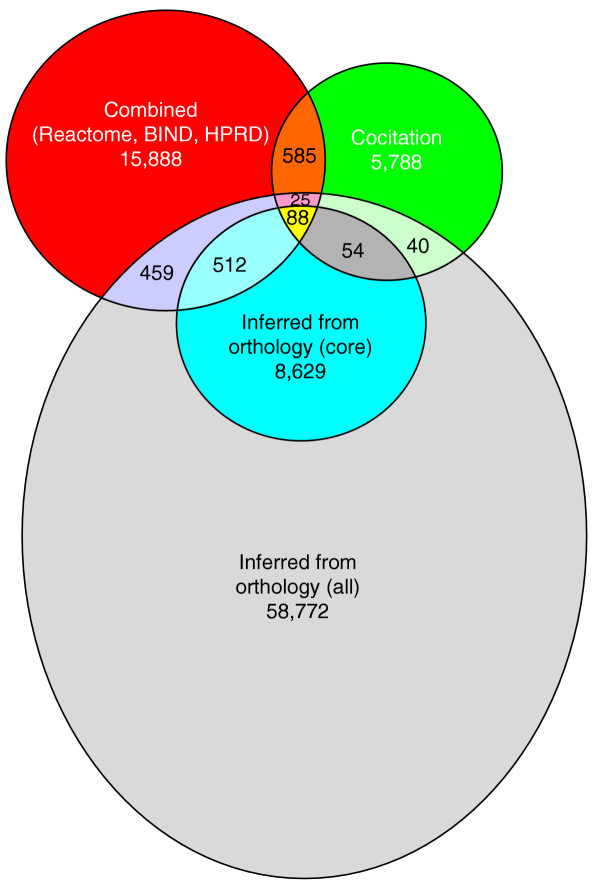
Comparison of extracted interactions with existing interactions. A comparison of interactions inferred from orthology [21] and those recovered by co-citation with the other existing human protein interaction datasets reveals that the overlap is small. The trend implies that the different methods are sampling relatively exclusive sets of interactions although, with the exception of the orthology-derived interactions, they are all derived directly from the primary biological literature.

Taking as a minimally acceptable level of accuracy the interactions hand-entered from Medline (HPRD), our co-citation/Bayesian classifier analysis yields 6,580 interactions between 3,737 proteins. By combining these interactions with the 26,280 interactions from other sources, we obtained a final set of 31,609 interactions between 7,748 human proteins. In this, we have chosen not to include the complete set of orthology-derived interactions due to their lower performance on the annotation benchmark, although these will ultimately be quite useful when supported by future data. Table 2 shows the contributions from each of the datasets at this threshold and a comparison of the overlap of interactions in each of the datasets is depicted as a Venn diagram in Figure 5. The Venn diagram indicates small overlap among the various datasets, with less than 0.2% of the interactions represented in all datasets. Nonetheless, this network of interactions represents the current state of the human interactome at a reasonable level of accuracy.

### The ID-Serve database of annotation and interactions

We have incorporated the results of this analysis into a web-based server [37], which can be queried for interactions of specific proteins. Genes are cross-listed under a variety of naming conventions, including LocusLink/EntrezGene, RefSeq, and Swiss-Prot, and are accompanied by links to other databases and GO and KEGG functional annotations. Protein interactions derived from the co-citation/Bayesian analysis are hyperlinked to the co-citing Medline abstracts, where they can be directly manually verified.

## Discussion

### Features of the network

**Figure 6:**
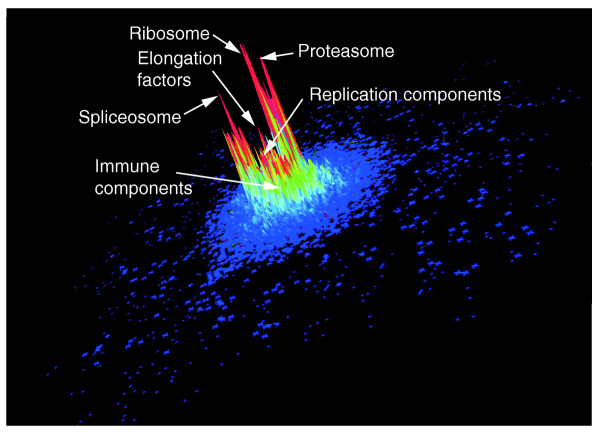
Visualization of the final consolidated network of protein interactions. A view of the composite interaction network (31,609 interactions between 7,748 proteins). Of these, 6,706 proteins (87%) are connected by at least one interaction into the central, connected network component. The modularity in the network can be seen in the superimposed three-dimensional visualization, a histogram in which higher peaks correspond to larger numbers of edges per unit area. The network coordinates were generated by LGL [46] and visualized with Zlab by Zack Simpson.

In order to study the features of the network, we visualized the complete network of protein interactions in Figure 6. On superimposing a histogram of the density of interactions on the plot, we see that there is considerable clustering of proteins in the network, represented as peaks in the histogram. A closer look reveals that these regions correspond to proteins involved with the ribosome, spliceosome, proteasome, replication, transcription and the immune components.

A quantitative analysis of the network clustering and connectivity distribution (reviewed in Barabasi and Oltvai [38]) is presented in Table 2. The clustering coefficient (<C>) captures the modularity of the network. A comparison of our final network (<C> = 0.24) with 10 randomly generated networks with the same number of interactions and proteins (<C> = 9 × 10^-3 ^± 3 × 10^-5^) shows the clustering in the human protein interaction network is considerably above that expected at random, in spite of the incompleteness of the network. The 'degree' of the network is defined as the average number of links per protein and captures the connectivity of the network. Except for Reactome, each of the datasets indicated in Table 2 show low connectivity. The combined network is intermediate in both connectivity and modularity. Projecting from the approximately 15 interactions per protein in the best sampled interaction dataset (Reactome) to the 25,000 or so estimated in the human genome [39] implies more than 375,000 interactions in the complete human protein interaction network. Note that any overestimates in the average number of interactions per protein will be counterbalanced by the effect of alternative splicing in increasing the number of actual proteins, making this estimate at least a reasonable ballpark estimate. The current set of interactions therefore represents no more than 10% of the complete network.

### Advantages of the log likelihood benchmarks

A good accuracy measure is of tremendous importance, impacting on the reliability of all downstream analysis. The log likelihood analysis eases comparison and assessment of diverse datasets. The score indicates the probability that the identified interactions are correct based on enrichment of positive interactions over background expectations. Note that this approach is distinct from simply measuring the intersection with the benchmark associations - because enrichment of positive to negative associations is measured, rather than just recovery of positive associations, even datasets with small intersections to the benchmark set can be evaluated for accuracy. Note also that the benchmarks themselves are not likely to be 100% correct - protein annotations are subjectively assigned, many proteins belong to multiple pathways, and even hand-curated protein interaction data can be mis-entered. Nonetheless, the log likelihood framework is tolerant of errors and merely requires that the benchmark data are generally correct among true interaction partners. Figure 4a shows the accuracy of each of the datasets. While the existing datasets have a single accuracy value, the mined interactions can be adjusted for accuracy based on the CRF threshold and the co-citation probabilities. New datasets can be incorporated using the log likelihood scoring scheme, and the ultimate strength of these benchmarks will be their utility in integrating data from diverse experiments [14].

### Shortcomings and strengths of literature mining via the co-citation/Bayesian classifier approach

From our previous work [32], we realized that directly identifying protein interactions would be a difficult task if we were unable to differentiate proteins and genes from the rest of the text. We therefore concentrated on building protein name extractors and interaction extractors in parallel so that the results of the former analysis could be fed into the latter.

Crucial to this process was the creation of a high-quality dictionary of human protein names and synonyms with mappings back to database entries. We therefore decided to start by creating a set of unambiguous gene names along with their synonyms that could all be mapped to a single unified gene identifier (LocusLink identifiers, now maintained through EntrezGene). The dictionary had to have very few spurious entries to ensure minimal false positives. The resulting ID-Serve database captures the various identifiers for a given gene and creates a repository for the retrieval of these genes along with their mined interactions. Building on this dictionary, the CRF algorithm then analyzed the context in which likely protein names appeared in order to identify the protein names more accurately. In the approach we describe, protein interaction partners are identified from among these protein names by a filtered version of co-citation.

The co-citation approach [14,26,40] calculates the random probability of co-occurrence of two protein names. The assumption is that if the co-citation is statistically unlikely under the random model, then there is a true underlying reason for the proteins to be co-cited - that is, they are interacting at either the functional, pathway level, or are co-localized or physically interact. The method has both advantages and disadvantages. It does not extract all interactions, but only those with statistically significant co-citations. By using the Bayesian estimator [36] we enrich further for physical interactions, but at the expense of coverage. Among the disadvantages are that the algorithm enriches for certain types of errors (for example, 'A does not interact with B', dictionary errors leading to synonyms being wrongly enriched, and so on). However, we feel the advantages outweigh the disadvantages: In particular, the probabilistic ranking, combined with the Bayesian filter, minimizes systematic errors, and at the left side of Figure 4b, it can be seen that errors in the co-citation data are no more extensive than errors introduced in transferring annotation from other organisms, or those errors introduced by human curators reading Medline abstracts. The method is easily applied, and currently outperforms other publicly available protein interaction extraction algorithms [34,35]. Finally, the precise nature of the interaction can be directly checked from the linked Medline abstracts. Thus, the mined interactions will be ideal for manual validation by curators of protein interaction databases (for example, DIP and BIND).

## Conclusion

In conclusion, to prepare for attempts to map the set of human protein interactions we sought to consolidate known interactions and to establish measures of accuracy that are useful for the evaluation and integration of upcoming datasets. We established two benchmarks for assessing the quality of large-scale human protein interaction datasets, providing quantitative measures useful for the testing and integration of interaction data. Using these benchmarks, along with available and mined interactions, we assembled an integrated dataset of 31,609 interactions between 7,748 human proteins, forming a framework for the interpretation of human functional genomics data. These data are collected in the ID-Serve database [37], which can be queried for protein interactions and their corresponding Medline citations. We estimate these interactions form less than 10% of the human interactome, setting the stage for future efforts to map the complete human network of protein interactions.

## Materials and methods

### Identification of human protein names and interactions in Medline abstracts

The training datasets used for the literature mining are as in [32]. The dictionary of human protein names was assembled from the LocusLink and Swiss-Prot databases by manually curating the gene names and synonyms (87,723 synonyms between 18,879 unique gene names) to remove genes that were referred to as 'hypothetical' or 'probable' and to omit entries that referred to more than one protein identifier. From the Medline database of approximately 11 million abstracts (1951-2002) we retrieved 753,459 abstracts containing the word 'human' either in the title or the text to use as our corpus for extracting protein interactions.

We have previously described [32] effective protein and gene name tagging using an algorithm based on maximum entropy. Conditional random fields (CRF) [41] are new types of probabilistic models that preserve all the advantages of maximum entropy models and at the same time avoid the label bias problem by allowing a sequence of tagging decisions to compete against each other in a global probabilistic model. In this paper, we show that CRF outperforms our best previous maximum entropy tagger.

In both training and testing the CRF protein-name tagger, the corresponding Medline abstracts were processed as follows: text was tokenized using white space as delimiters and treating all punctuation marks as separate tokens. The text was segmented into sentences, and part-of-speech tags were assigned to each token using Brill's tagger [42]. For each token in each sentence, a vector of binary features was generated using the feature templates employed by the maximum entropy approach described in [32]. Each feature occurring in the training data was associated with a parameter in the CRF model. We used the CRF implementation from McCallum [43]. To train the CRF's parameters, we used 750 Medline abstracts manually annotated for protein names [32]. We then tagged predicted protein names in the entire set of 753,459 Medline abstracts using the version of the CRF algorithm that utilizes the dictionary as part of the learned model (Figure 2), and in this way linked each tagged name to a dictionary entry. The Medline abstracts with marked-up protein names are available on request.

The model assigns each candidate phrase a probability of being a protein name. We selected all names scoring higher than a given threshold (testing thresholds between 40% and 95%), retaining the proteins' LocusLink identifiers along with the PubMed identifiers (PMID) of the associated abstracts. The significance of co-citation of two protein names across a set of Medline abstracts was calculated from the hypergeometric distribution [14,26] as:

,

where:



and *N *equals the total number of abstracts, *n *of which cite the first protein, *m *cite the second protein, and *l *cite both.

The top-scoring 15,000 co-cited protein pairs were then re-ranked according to the tendency of the co-citing abstracts to discuss protein-protein interactions. Specifically, the likelihood of a co-citing abstract to discuss physical protein interactions was evaluated using the naive Bayesian classifier as described in [36], which scores Medline abstracts according to usage frequencies of discriminating words relating to protein-protein interactions. For each co-cited protein pair, we calculated the average of the scores of the co-citing Medline abstracts, then re-ranked the co-cited protein pairs by these average scores.

### Analysis of network properties

We evaluated the clustering of genes in an interaction network [38] by calculating the average clustering coefficient (<C>) of the *N *genes as:



where *C*_*i *_is the clustering coefficient of gene *i*, evaluated over the set of genes with at least two interactions and measured as the number of links, *n*, among the gene's *k *neighbors, divided by the number of maximum possible linkages, *k*(*k *-*1*)/2.

### Construction of the functional annotation benchmark

The specific GO and KEGG annotations for the functional benchmarks were downloaded from the Gene Ontology database [44] and the KEGG database [45]. Within the GO process annotation hierarchy (more strictly, a directed acyclic graph (DAG)), the number of distinct annotation terms is maximal at level 8, where the level is defined as the number of nestings from the root node (level 1), as given in the Gene Ontology DAG file [44]. KEGG functional annotations were constructed as the sets of numerical codes for the KEGG pathway diagrams associated with each gene. The functional annotation benchmark is composed of all pairs of human genes sharing annotation from either source (KEGG or GO). For training and testing sets, annotated genes were randomly assigned into two categories and associations were only considered between genes of the same category.

### The ID-Serve database

ID-Serve is a relational mySQL database of human proteins created to simplify comparison of datasets with differing protein identifiers. The database maps 42,232 LocusLink (now EntrezGene) identifiers to their corresponding Genecard, Swiss-Prot, Ensembl, OMIM, Unigene, NCBI GI codes and Accession numbers and to the GO and KEGG pathway annotations. Protein interaction data can be retrieved from ID-Serve, with co-citation derived interactions hyperlinked to the supporting Medline abstracts.

## Additional data files

The following additional data relevant to the analysis, training and testing carried out in this work are available with the online version of this paper and can also be obtained from the ID-Serve database [37]. Additional data files 1 and 2 contain tables of protein 'tagger' training sets. Additional data file 3 contains a dictionary of human protein names and synonyms indexed to LocusLink identifiers. Additional data file 4 contains the final set of 31,609 protein interactions between 7,748 proteins derived from this analysis. Additional data file 5 contains the final set of co-citation/Bayesian classifier-derived interactions with the PubMed identifiers of co-citing abstracts. Additional data file 6 contains the benchmark training set of functional annotations. Additional data file 7 contains the benchmark test set of functional annotations. Additional data file 8 contains the benchmark set of physical interactions. Additional data file 9 contains the discriminating word list used by the Bayesian classifier to estimate the likelihood of Medline abstracts to discuss protein interactions.

## Supplementary Material

Additional File 1 Training set of 200 Medline abstracts with all occurrences of protein names tagged Click here for file

Additional File 2 Training set of 750 Medline abstracts with all occurrences of protein names tagged Click here for file

Additional File 3 Dictionary of human protein names and synonyms indexed to LocusLink identifiers Click here for file

Additional File 4 Final set of 31,609 protein interactions between 7,748 proteins derived from this analysis Click here for file

Additional File 5 Final set of co-citation/Bayesian classifier-derived interactions with the PubMed identifiers of co-citing abstracts Click here for file

Additional File 6 Benchmark training set of functional annotations Click here for file

Additional File 7 Benchmark test set of functional annotations Click here for file

Additional File 8 Benchmark set of physical interactions Click here for file

Additional File 9 Discriminating word list used by the Bayesian classifier to estimate the likelihood of Medline abstracts to discuss protein interactions Click here for file

## Figures and Tables

**Table 1:** The initial list of the interactions and proteins represented in each of the existing human protein interaction datasets with total interactions, unique self-interactions and unique non-self interactions

Dataset	Version	Total interactions (number of proteins)	Unique self (A-A) interactions (number of proteins)	Unique (A-B) interactions (number of proteins)
Reactome	08/03/04	12,497 (6,257)	160 (160)	12,336 (807)
BIND	08/03/04	6,212 (5,412)	549 (549)	5,663 (4,762)
HPRD*	04/12/04	12,013 (4,122)	3,028 (3,028)	6,054 (2,747)
Orthology transfer (all)	03/31/04	71,497 (6,257)	373 (373)	71,124 (6,228)
Orthology transfer (core)	03/31/04	11,488 (3,918)	206 (206)	11,282 (3,863)

*Difficult to measure: HPRD records genes by their names, leading occasionally to entries for the same gene under different synonyms. The numbers reported are after mapping to LocusLink.

**Table 2:** A comparison of the contributions of each dataset to the composite human protein interaction map, with network properties of each of the datasets

Dataset	Version	Number of interactions	Number of proteins	Clustering <C>	Connectivity <#interactions/protein>
Reactome	08/03/04	9,987	619	0.74	15.4
BIND	08/03/04	1,536	1,212	0.1	1.3
HPRD	04/12/04	6,054	2,747	0.09	2.2
Orthology inferred (core)	03/31/04	9,283	3,469	0.13	2.7
Co-citation	This paper	6,580	3,737	0.3	1.8
Total	This paper	31,609	7,748	0.24	4.1

An analysis of network features (clustering coefficient [38] and degree of connectivity) of each of the datasets indicates low degree (<k>) for all except Reactome, which is by far the most densely sampled protein interaction dataset. The final combined network is modular in structure and shows extensive, non-random clustering of proteins as compared to randomly generated networks with equal numbers of proteins and interactions (<C> = 9 × 10^-3 ^± -3 × 10^-5^; average of 10 trials).
